# Controversials of Microscopic Colitis

**DOI:** 10.3389/fmed.2021.717438

**Published:** 2021-10-12

**Authors:** Emese Mihaly, Árpád Patai, Zsolt Tulassay

**Affiliations:** Gastroenterology Unit, Department of Internal Medicine and Hematology, Semmelweis University, Budapest, Hungary

**Keywords:** microscopic colitis, collagenous colitis, lymphocytic colitis, incomplete microscopic colitis, natural course

## Abstract

Microscopic colitis (MC) has become a disease with increased awareness due to the availability of new data about the pathogenesis, diagnosis and therapy of this disease. The incidence of MC is increasing, reaching the incidence of the inflammatory bowel disease (IBD) in some populations. However, some aspects of MC are still controversial. It is unknown whether the changes of microbiome play a role in the pathogenesis and what is in the background of the different subtypes of disease that can transform into each other. Is there a connection between MC and IBD or why the histological changes do not follow the clinical activity? We do not know what the etiology of the incomplete MC is, and what its natural course is. The association of MC with celiac disease is well-known- is there a common pathogenesis? The MC treatment is budesonide. Its effectiveness is high, but the relapse rate is high, as well. Why would biologics be effective in these cases when budesonide is not? This mini-review makes an attempt to summarize the data about MC and highlight that there are still unanswered questions in the pathogenesis, diagnosis and therapy of the disease, which can initiate further investigations in the future.

The name microscopic colitis (MC) refers to the fact that in this form of the disease, endoscopic examination reveals intact colonic mucosa and only histopathological examination of the biopsy specimen describes pathological features.

It was first described in 1976, when Lindstrom (1) presented the history of a middle-aged woman, who suffered from chronic watery diarrhea and abdominal pain. Despite of the regular colonoscopic picture, a subepithelial collagenous bundle was demonstrated histopathologically, which otherwise also develops in collagen sprue. Because of the similar histological signs, Lindstrom named the lesion collagenous colitis (CC). In 1980, Read et al. (2) noted a mild, non-typical inflammation in the mucosal biopsy taken during regular colonoscopy from a patient with the same medical history. He used the microscopic colitis (MC) term to describe this disease (2). Re-examining similar reports and data from Read and others, Kingham and Levison concluded that these abnormalities corresponded to the early stages of CC (3, 4). Lazenby, in 1989, analyzing a tissue sample taken during regular endoscopy of a patient with chronic diarrhea, found significant lymphocyte infiltration of the epithelium with inflammation of the lamina propria. He called the abnormality lymphocytic colitis (LC) (5). The hierarchy of names has changed over the years. Microscopic colitis has become the accepted term representing the two subtypes and forms of LC and CC. The two forms can be distinguished on the basis of histological differences. In 8–16% of cases of chronic, non-bloody diarrhea, if colonoscopy shows a regular macroscopic picture, MC is likely the etiology. It is mainly found in people over 60 years of age, more often in women. However, MC is also found in patients younger than 45 years. CC was even described in children (6). CC is 9 times more common in women than in men. The gender difference in LC is less profound. MC is becoming more common worldwide. The cumulative incidence is 4.1 cases per 100,000 persons/year in CC and 4.9 cases per 100,000 persons/year in LC (7).

The incidence of MC has reached the level of IBD in some populations and has even become more common than Crohn's disease (8). The increased recognition of the disease may be due to the increased availability of colonoscopy and the increased awareness and knowledge of gastroenterologists and pathologists. There are also ethnic differences in the prevalence of MC (9). It is more common in white populations and Jews than in non-white, East Asian and Hispanic populations. Evidence to date suggests that MC does not increase the risk of colorectal carcinoma (10, 11).

## Pathogenesis

The pathogenesis of MC development is not clear, and is probably a consequence of several factors. The characteristic histological abnormalities and clinical symptoms may develop in response to different influences (12, 13). Uncontrolled immune responses to various luminal and mucosal factors occur in genetically predisposed individuals. A role of autoimmune events is also likely that is supported for example by its association with celiac disease (13). MC is an immune-mediated aberration with primary involvement of the acquired immune system and cytotoxic responses. Immune-mediated factors, microbial agents, exogenous toxins are translocated from the intestinal lumen to the mucosal layers. The primary biological change that triggers the immuno-inflammatory cascade in MC is probably the decreased expression of claudin1 and occludin tight-junction proteins and the resulted increase of the intestinal permeability (14, 15).

In the intestinal mucosa of MC patients, an increased expression of diverse cytokines develops with the appearance of T-helper (Th)1, and cytotoxic T cells (Tc)1 or Th17 and Tc17, leading to increased expression of tumor necrosis factor (TNF) alpha, interferon-gamma and several interleukins (17, 21, 22, 23) (16–18) (Table 1). This molecular sequence of events promotes lymphocyte infiltration in the intestinal intraepithelial layer, inhibits the elimination of activated T cells, eosinophils, and also inhibits the caseation of macrophage activation, neurophage proliferation, leading to mucosal permeability and collagen deposition. Several proinflammatory cytokines, which are increased in MC, also contribute to the development of fibrosis (19). The increased expression of VEGF in the epithelium and the proliferation of inflammatory cells and fibroblasts are crucial factors for collagen deposition (20).

**Table 1:** Expression of citokines in Microscopic colitis.

**Cytokine**	**LC/CC**	**Effect**
TNFa	↑	Increases the innate immune response
IFNg	↑	Increases lymphocyte infiltration in the intestine, reduces intestinal barrier, activates macrophages
IL-1b	↑ CC only	Neutrophil recruitment; NOS induction
IL-6	↑	Neutrophil recruitment; NOS induction
IL-12	↑	Enhances IFNg production via mononuclear cells of the lamia propria
IL-15	↑	Increases IEL activity and enhances its proliferation
IL-17A	↑	TNFa IL-1; promotes IL-6 release; neutrophil recruitment, NOS induction, tight-junction enhancement; induction of antimicrobial proteins
IL-21	↑	Pleiotrop and proinflammatory effect
IL-22	↑	Induces TNFa and IL-8; stimulates myofibroblasts to produce collagen; associated with disease activity
IL-23	↑	TNFa, IL-1 and IL-6 induce, neutrophil recruitment. NOS induction
IL-37	↓	Maintenance of inflammation

Although many regulatory and executive processes are shared in LC and CC, their immunological properties as a whole are distinct, with different lymphocyte characteristics that result in different responses to luminal stimulation, pathological mucosal barrier function and HLA correlations. The changing cytokine profile leads to increased expression of TNF-alpha and various interleukins (21) (Table 1).

As MC is more common in post-menopausal women, hormones have been implicated in its pathogenesis. Burke et al. analyzed data from 275 post-menopausal women with MC (22). They categorized patients according to hormone replacement therapy and found that past hormone replacement increased the risk of MC by 1.95-fold (0.5% CI: 1.37–2.78) and current treatment by 2.64-fold (95% CI: 1.78–3.90). They also examined whether oral hormonal contraception in the medical history influenced the risk of MC. The magnitude of the increase in risk was found to be 1.57-fold (95% CI 1.16–2.13) (22).

Changes in the composition of the gut bacteria have become an important factor in recent years in the investigation of the pathogenesis of inflammatory bowel diseases. Different variations in both the composition and diversity of the microbiome have been identified, but their clear pathogenic role has not yet been confirmed. In active MC, reduction of Akkermansia species, among others, has been demonstrated (23). Morgan observed a significant difference in the gut microbiome composition in the active phase of MC patients and those in remission, which was reflected in the diversity of the microbiome and the degree of dysbiosis (24). The dysbiosis index was significantly higher in active MC than in those in remission, or those with chronic functional diarrhea and healthy subjects. However, the relative amount of Alistipes putredinis in the microbiome decreased in MC compared to the other study groups. The Alistipes species are butyrate-producing bacteria with anti-inflammatory properties and their abundance was reduced in newly developed childhood IBD, as well (25). Morgan's study suggests that dysbiosis, like in inflammatory bowel disease, is an important feature of the gut microbiome in MC (24). However, it has not been shown that this abnormality is a primary pathogenic factor, as it may also develop as a secondary phenomenon.

Looking at the genetic background, the data on the association with human leukocyte antigen (HLA) are conflicting, but there is significant overlap with coeliac disease (26). Data also suggest an association between polymorphisms in the interleukin (IL)-6 gene and MC. This polymorphism leads to increased IL-6 production, which is an effective promoter of inflammation and fibrosis. The incidence of celiac disease is 3.3% in MC compared with 0.4% incidence in controls based on a large prospective study. No association was proved between gluten intake and MC development.

Differential expression of matrix metalloproteinases (MMPs) may also play a role in the pathogenesis of CC (13). MMPs are important players in remodeling following inflammatory processes: their genetic polymorphism may predispose to CC. The MMP-9 gene carrying the GG allele increases the risk of CC (13). Abnormal activity of MMP9 may lead to impaired collagen degradation.

Of the risk factors for MC, drug side effects are the most important. The most common drugs that cause MC are summarized in Table 2. The detailed pathomechanism by which proton pump inhibitors (PPIs) cause MC are unknown. However, proton pumps are present not only in the mucosa of the stomach, but also in the colon, thus affecting the potassium turnover of the whole body. Inhibition of the proton pumps in the colonic mucosa affects the local electrolyte balance, equilibrate the fluid acidity, which also affects the immune processes of the colonic mucosa. Hypomagnesemia caused by PPI may be a consequence of impaired magnesium absorption. PPIs impair important elements of magnesium absorption (13), and pH variations impair the function of the channels and tight junctions. PPI also increases fibrosis-promoting factors such as TGF-beta, fibroblast growth factor 2 and collagen types III and IV (27).

**Table 2:** Drugs increasing the risk of MC.

Acarbose
Aspirin
PPI
NSAID
H2-receptor Blockers
SSRI
Ticlopidine
Carbamazepine
Flutamid
Lizinopril
Levodopa/benserazid
Statins

In CC, COX-2 levels are increased in colonic mucosa (28). Persistent inhibition of COX-2 promotes myofibroblast-associated intestinal fibrosis (29). Verhaegh et al. (30) demonstrated in a case-control study that current and recent use of NSAIDs, as well as SSRIs, are associated with an increased risk of MC, when compared to control population.

Smoking is another risk factor. Smoking increases the risk of CC (OR: 5.5; 95% CI 3.4–8.9), but is has less effect on the risk of LC (OR: 2.96; 95% CI, 2.0–4.3) (31).

A recently recognized risk factor is the *Campylobacter concisus* infection. Nielsen et al. (32) demonstrated in a population-based cohort study that the risk of MC is twice as high in patients after *C. concisus* infection compared to patients with negative stool culture. The risk is highest in the first year after the infection, but is also high for 9 years up. The correlation between the *C. concisus* infection and CC subtype was demonstrated.

Diarrhea in MC is a consequence of several factors. These include:

- osmosis
- reduced absorption: in LC, sodium channels of the colonic epithelium are inhibited (33), and levels of fecal, intestinal lumen nitric oxide and epithelial nitric oxide synthase are increased (34)
- active secretion of chloride in CC (14)
- abnormal epithelial barrier function (33)
- abnormal regulation of aquaporins
- abnormal motility (35)
- impaired absorption of bile acids (36).

## Symptoms, Natural Course

The leading symptom of MC is chronic, watery diarrhea, which may be associated with stool leakage. Nocturnal diarrhea can also be present. General symptoms may also include weight loss, fatigue, in rare cases, electrolyte disturbances and dehydration. After a temporary period of relief, diarrhea may return.

The natural course of MC is characterized by alternating periods of asymptomatic and diarrheal episodes. Symptoms may disappear for years without treatment. Symptoms are effectively controlled by budesonide, but after discontinuation of this treatment, symptoms return within 3 months in 80% of patients. Despite the recurrence of symptoms, the disease does not worsen, is not associated with an increased risk of mortality and malignant tumors of the colon (11).

## Diagnosis

The three determinants of the diagnosis are:

- characteristic clinical symptoms
- normal endoscopy picture of the colon
- pathognomic histological picture.

Symptoms and a regular macroscopic picture may be features of many pathologies that should be considered in the differential diagnosis (Table 3).

**Table 3:** Differential diagnosis of chronic diarrhea, when the endoscopic picture is normal.

Microscopic colitis
Celiac disease
Infection (eg. cryptosporidiosis)
SIBO
Giardiasis
Impaired bile acid absorption
Neuroendocrine tumors
Laxative abuse
Carbohydrate absorption disorders (e.g., lactose, sorbitol)
Irritable bowel syndrome

Neither laboratory abnormalities nor biomarkers (e.g., calprotectin) are of diagnostic value. A scoring system has also been developed to identify the pathology of MC, taking into account various risk factors and symptoms, but it is currently not a reliable guide (37).

The colonoscopic picture is mostly normal, although non-specific abnormalities (hyperemia, edema, decreased vascularity, patchy erythema) may occur (38).

The histological hallmark of LC is intraepithelial lymphocytosis. The criterion is at least 20 intraepithelial lymphocytes (IEL) out of 100 surface epithelial cells by hematoxylin-eosin staining. The normal value is not more than 5. The surface epithelium may be slightly damaged and little collagen is deposited in the subepithelium. If the number of IELs is borderline, immunohistochemistry is recommended to detect intraepithelial T cells, which is a more sensitive method, detecting IELs than hematoxylin-eosin staining, and therefore it is recommended, especially in borderline cases, when the diagnosis is difficult with routine staining.

The diagnosis of CC requires thickening of the collagen bundle beneath the superficial epithelium, which must exceed 10 μm. The superficial epithelium may also be damaged and the number of IELs cells may increase, but does not reach the level typical of LC. In the majority of cases hematoxylin-eosin staining is pathognomic, but in borderline cases connective tissue staining is also required.

In both histological forms, the epithelium is damaged, mucus is deposited, and vascularization is observed. Inflammatory infiltration develops in the lamina propria, with plasma cells, lymphocytes, eosinophils, mastocytes, sometimes with Paneth cell metaplasia and cryptitis.

In incomplete microscopic colitis (MCi), the pathological criteria for LC and CC are not fully met. Although the number of IELs cells and the width of the collagen bundle increase, they do not reach the pathological threshold (39–41). MCi is the same of MC in terms of the clinical symptoms, the nature of histological lesions, and in terms of treatment, as well. The only difference is quantity. This calls into question the validity of the MCi diagnosis. Quantitative differences may be due to the location of the biopsy, may depend on the quality of histological processing. The relationship between MCi and MC is also unclear. Can these transform into each other? If someone had an MCi, could they later have an MC, or vice versa? Determining the numerical limit is arbitrary. What explains this limit? Why this limit is as much as it is? Could it be more or less? If there is continuity between the incomplete and complete forms and the abnormality can regress, it may be that histological examination was performed at this stage.

The choice of the location and number of biopsies is essential for the diagnosis. According to previous statements, MC can develop in patches, and cellularity and collagen thickening vary in different locations in the colon, so biopsies taken from unaffected sections may be a source of error. There are no areas of distinction in different sections of the colon that may help to diagnose the pathology. Therefore, it was considered that a minimum of eight biopsies from each colon section is required for pathology (38, 42). Fiehn et al. (43), however, demonstrated that the histological changes of MC were more pronounced in the right colon, but the diagnostic histological criteria were also present in 90% of the left sided biopsies as well (rectum excluded), outlining that MC is a pancolitis and debated the previous patchy manifestation of the disease. Therefore, the number of biopsies can be reduced taking them only from the right and/or from the left colon.

## Treatment

### Budesonide

The first line of treatment for MC is budesonide, a second generation of corticosteroids, which is a topical agent that binds sensitively to intracellular glucocorticoid receptors at the site of inflammation. It has a significant (about 90%) first-pass metabolism in the small intestinal mucosa and liver and therefore has little overall systemic effect.

In his meta-analysis, Kafil processed data from four randomized placebo-controlled trials and showed that after 6–8 weeks of treatment, 81% of patients treated with budesonide improved, which was significantly different from the placebo group (36%) (44). Histological healing was also more frequent in the budesonide group (78 vs. 32%). Symptoms of watery diarrhea improved within 2 weeks. The 9 mg/day budesonide induction treatment was effective in both LC (45, 46) and CC (47, 48). However, when treatment is discontinued, symptoms return in 60–80% of patients, particularly in CC (49). Two randomized trials showed that the improvement was sustained with 6 mg of budesonide daily for 6 months in 75% of CC patients, compared with 25% in the placebo group (44). No controlled trial of maintenance treatment of LC has been conducted, but experience confirms the need.

Budesonide is an effective treatment in all types of MC. It is unclear, however, whether the treatment cures MC or at first it converts MC to MCi.

### Other Inflammation and Secretion Inhibitors

Prednisolone is less effective than budesonide (50, 51) and symptoms return more frequently after discontinuation of treatment. In Munck's placebo-controlled trial, prednisolone was found to be ineffective (52). Comparing the efficacy of mesalazine (M) with budesonide (B) and placebo (P), mesalazine was inferior to budesonide in both forms of MC (CC: B: 80%, M: 44%, P: 38%; LC: B: 79%, M: 63%, P: 42%) (53).

There have not been extensive trials with bismuth subsalicylate, but anecdotal evidence suggests that it is more effective than placebo. Loperamide has a beneficial effect on symptoms in mild MC, but no systematic studies have been conducted with this agent. If the diarrheal effect of bile acids in MC is also detected, cholestyramine has a good effect on symptoms (54).

### Immunomodulators

Cotter treated 49 patients with MC for an average of 4 months with thiopurine (55). He found complete response in 43 % of patients and partial response in 22 % of patients, while 17 patients discontinued the treatment due to severe side effects (55). A prospective study with methotrexate was performed in 9 patients who did not respond to budesonide or who did not tolerate this treatment. None of them experienced any improvement (56).

### Biological Treatment

No comparative studies with biological agents have been done in large numbers of patients. They are currently considered second-line agents, mainly when budesonide is ineffective. Among the TNF-alpha antagonists, infliximab and adalimumab have been the subject of case reports and papers describing the data of small number of patients. The largest number of patients was reported by Daferera (14 CC out of 16 MC, 2 LC) (57). In eight patients, the treatment resulted in clinical remission and 4 patients failed the therapy. Among the integrin inhibitors, vedolizumab treatment was reported in 11 patients (5 LC, 6 CC) (58) in whom no other agent was successful. Three infusions improved the condition of 5 patients (2 LC and 3 CC), and 3 remained in remission after 13 months of maintenance treatment. Several successful case reports are also known (59–61).

### Stool Transplantation

The first fecal microbiota transplantation (FMT) in CC was reported by Günaltay et al. (62). The treatment was justified by the ineffectiveness of budesonide. After three FMTs, the patient's symptoms resolved. Holster reported on FMT treatment of 10 CC patients (63). However, the indication for FMT is still uncertain and the results are still inconclusive.

### Recommendation

**Figure 1:**
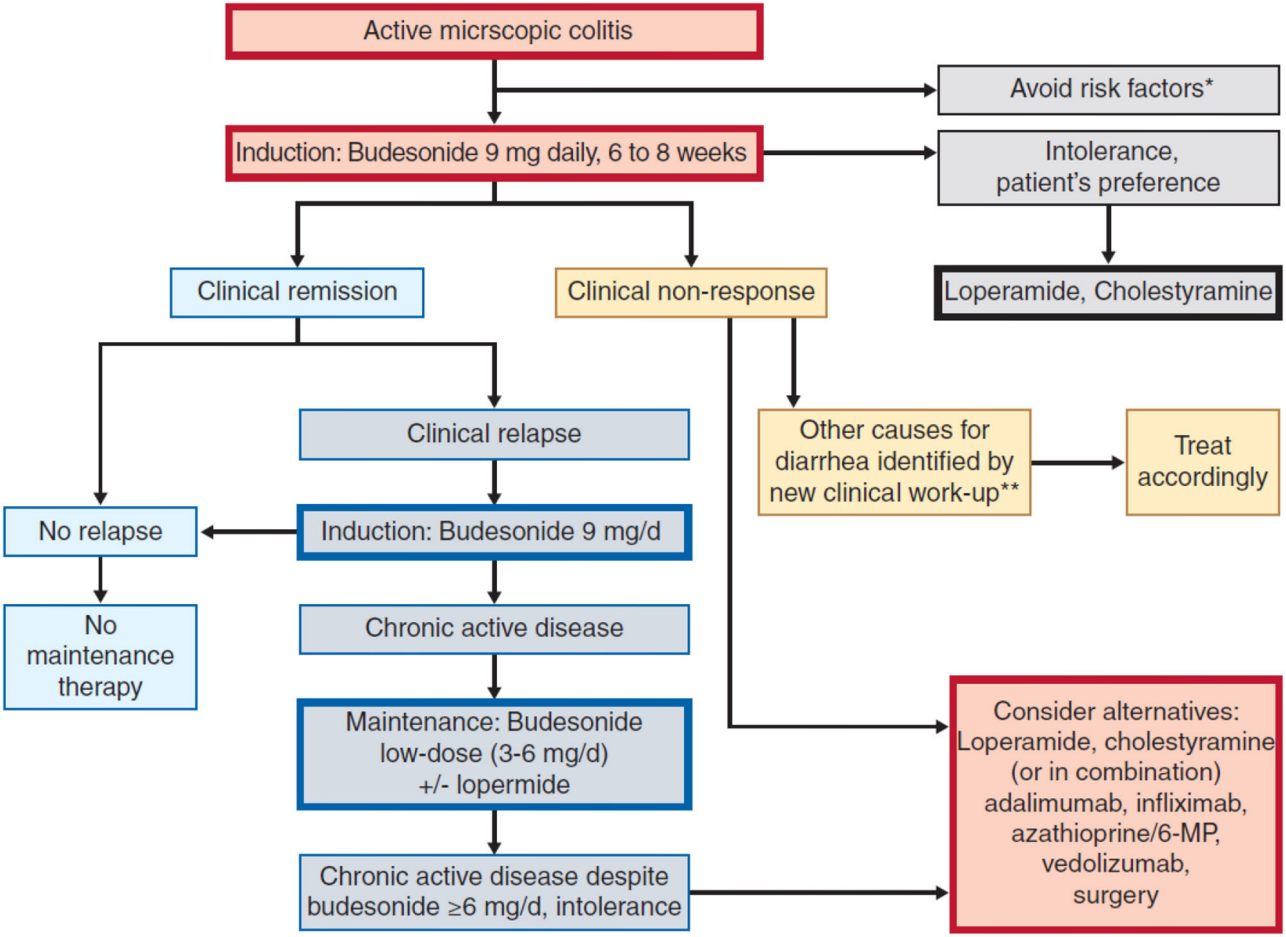
Therapeutic algorithm for Microsopic Colitis- Miehlke et al. (64). *Smoking, NSAID, PPl, **i.e., bile acid diarrhoea, coeliac disease.

The most effective treatment for active MC is budesonide, which is the only product registered for this indication. In chronic disease states, maintenance treatment with reduced dose is indicated. Other treatments are considered only as second-line treatments when budesonide is ineffective (Figure 1). When drug induced MC is presumed, withdrawal of the suspected drug/drugs is suggested by European guidelines on MC (strong consensus, low level of evidence).

## Directions of the Cryptic Pathways

Despite the growing knowledge on MC, there is still much to be discovered (62). Unanswered questions are the classification, the pathology, its relationship to inflammatory bowel disease, whether it is truly a distinct entity or whether it is a response to various influences and an intermediate state whose direction of development is the result of the interaction of many factors. One basis of uncertainty is the fact that forms of MC can transform into each other and into classical inflammatory bowel disease, and that genetic similarities with IBD can be detected.

The two forms of MC can be distinguished from each other by histological signs. The definition of a so-called incomplete variant (MCi) is also supported by criteria that seem clear. On this basis, the view that LC and CC are two different manifestations of the same disease cannot be challenged. However, longer follow-up of patients with MC has revealed a remarkable association that needs to be explained. Rasmussen et al. (42) performed repeated colonoscopic and histological examinations of 283 patients (149 (32%) CC, 72 (20%) LC, and 62 patients (27%) MCi) out of 1,055 patients with MC (468 CC; 361 LC; 226 MCi). The examinations were performed within 12 months, between 13–24 and 24 months after the diagnosis. Histological pathology was unchanged in 83% of CC patients, 63% of LC and 54% of MCi patients. However, the abnormalities characteristic of each form were changed in 107 patients (26%): the previously established pathology was replaced by features of the other MC form (CC: 22; LC: 39; MCi: 44 patients). CC pathology changed to LC in 13 patients and to MCi in 9. The trend of LC conversion was: to CC: 31, to MCi: 8. The change of MCi: 30 to CC and 14 to LC.

Vigren reviewed colonoscopic and histological findings of 65 patients with MC (CC: 47; LC: 18) at least 3 months apart (65). The follow-up demonstrated transformation of the pathology in 9 patients: in three patients CC turned into LC and in six patients, LC turned into CC. In 17 of the 47 CC cases, the histopathological picture became normal, and in 27 the MC did not change. In four of the LC patients, the histology remained unchanged, while in eight, a regular structure was detected.

Ung's repeated the endoscopy of 23 patients with LC after 4 years and showed the appearance of CC in two of them (66). Olesen checked 25 patients with LC 2 years later and found that nine had normal histology, 11 still had LC and five had CC (67).

The background to the transformation of each form is unknown. Vigren also reviewed the way the patients were treated with medication, assuming that the change or transformation was a consequence of the treatment. However, he did not find a correlation between the drugs and the transformation of the histological picture (65).

When evaluating the results of a control colonoscopy in MC, the possibility of sampling error should be taken into account. The collagen layer may also thicken in patches, alternating with a normal-sized mucosal structure. Few biopsy samples may therefore be a source of error. To avoid the possibility of error due to sample number in the pathology of MC, two biopsies from each segment of the colon are recommended. The method of sampling also influences the certainty of the pathology. Biopsies taken only tangentially to the mucosa are not suitable for determining the thickness of the subepithelial collagen layer. Histological sampling of the area of proliferating lymphoid follicles, in which many intraepithelial lymphocytes are visible, may lead to a misdiagnosis of LC. However, this is only a local phenomenon and does not apply to the colonic mucosa as a whole.

The rate of CC transformation is lower than that of LC, which can be explained by several possibilities. CC is a more active and stable form of the disease, as the collagen bundle formed is less reversible than the change in the number of lymphocytes proliferating intraepithelially. They are more mobile and their numbers may fluctuate as they migrate into the epithelium.

Incomplete MC transforms most commonly, that may be explained by the fact that MCi may be the initial form of MC and tissue abnormalities may be observed in the proliferation of collagen and intraepithelial lymphocytes, although not pathognomonic. Depending on the triggers of the disease process, the tissue structure may be of either form.

Histological changes may also raise new aspects in the assessment of MC:

- The clinical picture may also change during the natural course of the disease, which could be considered a characteristic feature of MC. The data available to date are insufficient to prove this possibility. Systematization of homogeneous data may help to resolve this issue.
- The location and processing of histological sampling may also be a source of error. Biopsies from different sites and treated with different staining procedures may reveal different tissue structures.

Histopathological analyses suggest that classical inflammatory bowel diseases may be associated with histological abnormalities of microscopic colitis at the beginning or at certain stages of the disease process (68). There are case reports that CC may progress to severe ulcerative colitis (69, 70) or Crohn's disease (71).

On the association between inflammatory bowel disease and MC, Khalili et al. reported summary data from a 27-year prospective study in Sweden (72). During their 27 years of follow-up, 13,957 MC cases were reported in 28 gastroenterology centers in Sweden. Data from MC patients were compared with the healthy general population (*n* = 66,820). In MC patients, 323 UC and 108 CD developed during the follow-up period. The incidence of IBD in the healthy population was significantly lower (UC: 94; CD: 42). On average, IBD developed in the third year of MC. Khalili's results show that MC increases the relative risk of IBD by 17-fold. Based on smaller number of patients and shorter follow-up periods, other authors have not demonstrated this association (73, 74).

Although the exact pathogenesis of IBD and MC is not known, their pathogenesis shows several common factors (75).

- An association between HLA haplotypes DQ2 and DQ8 and the risk of MC is known (76). Westerlind has demonstrated a significant overlap in single nucleotide polymorphisms (SNPs) associated to either IBD or MC phenotypes (77). Green's genome-wide association study (GWAS) showed an association with haplotype 8.1, suggesting the importance of an immune component in the development of MC (26). Genetic risk score calculations show a similar trend between MC and Crohn's disease, but not with ulcerative colitis.

Green's data confirm the association between the phenotype of MC and celiac disease, which may further explain the frequent co-occurrence of the two conditions (78).

- The role of the microbiome, significant dysbiosis and reduced diversity in the pathogenesis of IBD and MC is increasingly recognized.
- Histopathological images of lymphocytic colitis also show non-squamous granulomas and impaired crypts structure, similar to those observed in Crohn's disease (79).
- In MC, the expression of proinflammatory cytokines (e.g., interferon-gamma, tumor necrosis factor-alpha, IL-17, etc.) is increased in the mucosal immune response (Table 1), which is also observed in inflammatory bowel disease. This suggests that MC could be an early form of IBD, in which mucosal inflammation is attenuated and balanced by the anti-inflammatory effect of IL-10. When this counteracting effect is reduced or eliminated, Th1 and inflammatory Th17 cells proliferate and the classic inflammatory bowel disease (IBD) may develop.

Several details of the pathogenesis of inflammatory bowel disease are known, highlighting the role of different factors and suggesting the likelihood of a number of correlations. However, the new data are heterogeneous and hinder the development of a unified view and understanding. This is particularly true for microscopic colitis, where the findings often raise doubts and lead to new research targets. Much is known about MC, and its classification and criteria are largely within acceptable limits. However, doubts, contradictory views and facts are increasingly pointing in the direction of a number of hidden pathways, which may be a task for the future.

## Author Contributions

EM wrote the first draft of the manuscript, contributed to conception, and design of the study. ZT contributed to conception and design of the study. ÁP is a member of the Microscopic study group in our Department. All authors contributed to manuscript revision, read, and approved the submitted version.

## Conflict of Interest

The authors declare that the research was conducted in the absence of any commercial or financial relationships that could be construed as a potential conflict of interest.

## Publisher's Note

All claims expressed in this article are solely those of the authors and do not necessarily represent those of their affiliated organizations, or those of the publisher, the editors and the reviewers. Any product that may be evaluated in this article, or claim that may be made by its manufacturer, is not guaranteed or endorsed by the publisher.
